# Differences Related to Cancer Screening by Minority and Rural/Urban Status in the Deep South: Population-based Survey Results

**DOI:** 10.7150/jca.49676

**Published:** 2021-01-01

**Authors:** Casey Daniel, Salma Aly, Sejong Bae, Isabel Scarinci, Claudia Hardy, Mona Fouad, Wendy Demark-Wahnefried

**Affiliations:** 1 Faculty of Medicine, Alexandria University, Alexandria, Egypt.; 2 O'Neal Comprehensive Cancer Center at the University of Alabama at Birmingham (UAB), Birmingham, AL.; 3 University of South Alabama College of Medicine.

**Keywords:** cancer screening, race, rural, mammography, colonoscopy

## Abstract

**Objective:** Cancer mortality in the U.S. Deep South exceeds national levels. A cross-sectional survey was undertaken across Alabama to discern cancer beliefs and screening practices, and compare data from racial/ethnic minority versus majority and rural versus urban respondents.

**Methods:** Using population-based methods, we approached 5,633 Alabamians (ages 50-80) to complete a 58-item survey (administered in-person, via telephone, or the web). Descriptive statistics were used to summarize findings; two-tailed, chi-square and t-tests (α<0.05) were used to compare minority-majority and rural-urban subgroups.

**Results:** The response rate was 15.2%; respondents identified as minority (n=356) or majority (n=486), and rural (n=671) or urban (n=183). Mean (SD) age was 63.7 (10.2) and >90% indicated stable housing, and healthcare coverage and access. Rural and minority versus urban and majority respondents were significantly more likely to have lower education, employment, and income, respectively. Most respondents equated cancer as a “death sentence” and were unable to identify the age at which cancer screening should begin. Few rural-urban subgroup differences were noted, though significant differences were observed between minority versus majority subgroups for mammography (36.7% versus 49.6%, p<.001) and colorectal cancer screening (34.5% vs. 47.9%, *p*<0.001). Furthermore, while minorities were significantly more likely to report ever having a colonoscopy (82.1% versus 76.1%, *p*=0.041) and to have received fecal occult blood testing within the past year (17.2% versus 12.2%, *p*=0.046), routine adherence to screening was <20% across all subgroups.

**Discussion:** Cancer early detection education is needed across Alabama to improve cancer screening, and particularly needed among racial/ethnic minorities to raise cancer awareness.

## Introduction

Cancer remains the second leading cause of death in the United States and internationally, though rates have declined in recent decades 1, 2. These decreasing cancer mortality rates are largely attributed to advancements in screening and early detection of many cancers, leading to earlier diagnosis and more effective treatment 3. However, despite the overall decline, cancer rates vary substantially within the U.S. with regions such as the Deep South, indicating disproportionately higher rates compared to other areas 4. These disparities are well-documented in Alabama where cancer incidence and mortality remain higher than national levels, i.e., 448.0 *versus* 443.6 per 100,000, and 184.4 *versus* 166.1 per 100,000, respectively 4. In addition to regional discrepancies, racial and ethnic disparities in cancer mortality are well-established. While biologic differences and lifestyle and behavioral factors account for some observed racial/ethnic differences in cancer mortality, barriers to healthcare and/or inadequate quality healthcare are among the most significant contributors to these disparities 5. Limited access to care frequently translates to gaps in cancer early detection services. Lacking awareness of and/or access to recommended, regular screening may cause delays in diagnosis and more advanced disease requiring more invasive treatment as well as lower rates of successful recovery and potentially poorer prognosis 5. Such racial disparities are of particular concern in Alabama where African Americans, who comprise 26.3% of the state's population, have markedly higher cancer-related mortality in a state already outranking the national average 4, 6.

Rurality is another likely contributor to Alabama's increased cancer mortality since 55 (82%) of its 67 counties are classified as rural, as determined by: (1) the percentage of total employment in the county comprised by those employed by the public and secondary school systems, (2) the dollar value of agricultural production per square mile of land, (3) the population per square mile of land, and (4) an index used to assign a score to counties which takes into consideration the population of each county's largest city, its other cities, and its cities which are in more than one county 7. Compared to their urban counterparts, rural populations have increased rates of preventable and chronic conditions, such as obesity, diabetes, and cancer 8-10. Individuals in rural settings also face more barriers leading to negative health outcomes including decreased access to healthcare, provider shortages, higher poverty, and increased rates of uninsured 11, 12. In a study assessing national socioeconomic and racial disparity trends in cancer incidence and mortality, Singh et al. found that those in more isolated settings or with lower education and income had higher mortality, particularly for lung, colorectal, and cervical cancers 13. This may be explained, in part, by previous findings that rural communities demonstrate lower uptake of primary preventive behaviors such human papillomavirus vaccination, and secondary preventive behaviors such as screening 14, 15. Previous studies have determined associations between common rural factors such as poverty and lower screening participation, making this an important area for potential intervention 16, 17. This may be especially true for Alabama where three of the cancers with the greatest incidence and/or mortality-breast, colorectal, and lung-benefit tremendously from early detection 18-21.

Previous work performed by the Deep South Network for Cancer Control to improve cancer screening has demonstrated success 22. However, more efforts are needed to address the needs of specific communities to reduce high rates of cancer mortality through improved, targeted interventions emphasizing increased access to care and early detection. The aim of the current study was to determine cancer beliefs and screening practices among Alabama residents, comparing data from racial/ethnic minority versus majority respondents as well as rural versus urban respondents.

## Methods

This exploratory, cross-sectional survey study was initiated as part of the National Cancer Institute (NCI) Population Health Assessment in Cancer Center Catchment Areas Rural Workgroup - Round 2 and approved by the University of Alabama at Birmingham Institutional Review Board (IRB-300002366). Surveys were administered between April and November 2019.

A probability-based sampling plan was employed using a direct mail database with coverage across Alabama. In an effort to focus on the 34 (out of 67) counties in which the incidence and mortality rates of cancer were higher, we doubly-sampled individuals residing in high-risk versus lower-risk counties, making sure that gender distribution was even and the sample was representative in terms of race and ethnicity with population estimates. At the time of the survey, 12 of the 67 counties in Alabama were recognized as urban and the remaining 55 were rural; there was an even distribution of high- and low-risk counties within each of these sectors.

Letters of invitation (n=5,633) to participate in the survey either via the web (with provision of a link) or by telephone (with provision of a toll-free number to volunteer or opt-out) were posted to residents of all counties (Clay and Dallas counties being an exception since an intensive face-to-face cancer prevention and control effort was underway that allowed for paper surveys to be interviewer-administered given our physical presence in the county). Addresses and phone numbers were secured through a commercial vendor that gathers information through roughly 25 different sources (Caldwell List Company, Roswell, GA). Given the survey's focus on cancer screening, mailings were directed toward county residents who were 50-80 years of age. Individuals not opting-out or not completing the survey within two weeks were telephoned up to six times. Given substantial numbers of letters returned as undeliverable and non-working telephone numbers, contact was only considered viable if the following conditions were met: 1) the letter was not returned as undeliverable; 2) a working telephone number could be reached (albeit not always answered); and 3) addressees were not found to have moved and/or have a medical condition that precluded survey completion and/or deceased. Respondents received $5 for completing web- or paper-based surveys (which were less costly to administer) and $2 for completing the telephone survey.

### Survey

The 58-item survey was comprised of all items that were recommended by the NCI Population Health Assessment in Cancer Center Catchment Areas Rural Workgroup - Round 2, and therefore included validated items from the Health Information National Trends Survey, the Behavioral Risk Factor Surveillance System Survey (BRFSS), and the National Health Interview Survey and solicited information on demographics, and cancer knowledge, beliefs, and screening practices 23-25. Items on cancer screening focused on both the receipt and timing of colorectal cancer (fecal occult blood testing [FOBT] and colonoscopy) and breast cancer (mammography) screening. All respondents were asked general questions on screening, such as the recommended age for screening initiation; however, screening practices were only assessed on individuals who were of the appropriate age and sex. The U.S. Preventive Services Task Force (USPTF) guidelines were used as the referent. For example, regarding mammography screening, USPTF guidelines recommend that women ages 50-74 receive biennial mammograms; therefore, respondents indicating that age 50 was the appropriate age to start screening were categorized as correct (as were respondents who indicated earlier ages among higher risk women). Only responses of women within that age range who indicated screening mammography were analyzed.

### Statistical Analysis

#### Sample Size/Power

The original power calculations were based on attaining a total sample size of 1957 with proportions of minority: majority being 27%: 73% and rural: urban 67%: 33%. This would provide 80% power at a 0.05 significance level using a two-sided z test to detect minimum differences of 0.13-0.14 or differences that are considered small as defined by Cohen 26. Having achieved a sample size of 855, we have 80% power at a 0.05 significance level using a two-sided z test to detect differences of 0.20-0.23 or of the magnitude considered small to medium as defined by Cohen 26.

#### Analysis Plan

For this exploratory cross-sectional study, measures of central tendency (e.g., means, medians) and variation (standard deviations) were used to summarize the data, and missing data were excluded from analyses. Differences between subgroups were tested using chi-square (dichotomous data) and two sample *t*-tests (continuous data) at α<0.05. Analyses were performed using the Statistical Package for Social Sciences (SPSS, version 24, IBM, Armonk, NY).

## Results

A total of 5,633 residents of Alabama were contacted for this survey and 855 completed it, yielding a response rate of 15.2%. **Table 1** provides socio-demographic, health, and access characteristics on the sample overall, as well as by minority-majority, and rural-urban status.

Overall, slightly more than half of the sample was female, married (or stable union), and reported educational attainment beyond high school. Average age was 64 years and one-quarter of the sample reported a previous cancer diagnosis. Respondents were fairly evenly distributed in terms of employment status, with roughly one-third being employed, one-third retired and one-third disabled/unemployed. Likewise, about one-third of the sample reported annual household incomes less than $20K and one-third had incomes of $75K or more. Despite this, roughly half reported “living comfortably,” and most conveyed stable housing, food security, healthcare coverage, and access to healthcare and the internet. Given our sampling framework which targeted counties with increased cancer burden coupled by the fact that most counties in Alabama are rural, 42% of respondents were racial/ethnic minorities and 79% resided in a rural-designated county. Of note, rural-urban status did not differ between majority and minority subgroups; however, there were a significantly higher proportion of African-Americans among rural respondents as compared to urban counterparts.

Subgroup comparisons between minority-majority and rural-urban residents revealed that the sample was similar in terms of age, stable housing, and the presence of at least some form(s) of healthcare coverage and access. However, both racial/ethnic minorities, as compared to non-minorities, and rural county residents, as compared to urban county residents, reported significantly lower educational attainment, employment, income, food security, and internet access. Significant differences in perceived income adequacy also were found between rural-urban respondents, but not between minority-majority subgroups; however, here several significant differences were detected with racial/ethnic minority respondents less likely to be female or to report marital status as married or stable union, private insurance coverage, healthcare access through doctor's offices or clinics, and previous cancer diagnoses.

**Table 2** provides data on cancer beliefs, knowledge and screening practices. Here, few significant rural-urban differences were noted, except that rural residents were far more likely to acknowledge the role of family history in portending future cancer risk and considered themselves at higher risk than their urban counterparts. In contrast, several significant differences were noted between minority-majority subgroups. In terms of cancer beliefs, racial/ethnic minority as compared to non-minority respondents, were more likely to view “cancer as a death sentence,” but expressed more of desire to know whether or not they had cancer, and assessed their personal cancer risk as relatively low. With the exception of awareness of spiral CT as a screening test for lung cancer, racial/ethnic minority respondents were less likely to correctly identify guideline ages for which colorectal cancer and breast cancer screening are recommended to begin and were far less likely to attribute family history as a risk factor for cancer. Additionally, a significantly higher percentage of minority versus majority respondents reported having an FOBT within the past year and ever having a colonoscopy. In the sample overall however, it was clear that a majority of respondents were unable to identify the ages at which breast and colorectal cancer screening are recommended. Furthermore, despite the fact that most reported receipt of a colonoscopy or a mammogram, far fewer adhered to guidelines regarding the frequency of screening, as indicated by percentages lower than 20% “across-the-board” for receipt of FOBT within the past year, colonoscopy within the past 10 years or mammography within the past 2 years.

## Discussion

Given that the state's cancer mortality exceeds national rates, this study endeavored to assess Alabamians' cancer beliefs and screening practices, particularly examining differences between minority-majority respondents and rural-urban respondents. Despite a fairly large sample size, few statistically significant differences were observed between rural and urban participants overall. In prior studies that report differences, such as that by Tran & Tran who found an Average Adjusted Prediction (AAP) of 93.5% versus 94.6% in rural versus urban sectors, respectively, a sample size that was 500-fold that of our study (i.e., 482,360) was required 27. Data on rural-urban status in other areas of cancer screening are largely absent from the literature, thus making our data related to colorectal screening one of the first reports. We also found that rural individuals were much more likely than their urban counterparts to acknowledge family history as a risk factor for future cancer risk and also considered themselves to be at greater risk of developing cancer than urban residents. Relatedly, Llanos et al. reported fairly high levels of cancer worry in Appalachian populations; however, also made the observation that cancer worry was unrelated to the uptake of colorectal cancer screening 28. This finding may explain why we did not observe rural-urban differences in cancer screening.

In contrast to rural-urban comparisons, several significant differences were found between racial/ethnic minority versus majority subgroups. Compared to their majority counterparts, racial/ethnic minority respondents were more likely to associate cancer with death. This finding is unsurprising given numerous studies that have reported fatalistic attitudes regarding cancer among African Americans 29-32. These widespread attitudes and cancer-associated fears have been linked to African Americans being less likely to seek screening, leading to advanced disease at diagnosis, worse health outcomes, and higher cancer mortality. Despite this, racial/ethnic minority participants in the current sample expressed a greater desire to know of their cancer risk, perhaps because many indicated fairly low perceived susceptibility to cancer compared to their non-minority peers. Other studies have demonstrated similar findings, indicating a belief among some African Americans that Whites are at greater risk of developing cancer 32, 33. Other reasons that African Americans may cite low perceived cancer risk include perceptions that they lack behavioral risk factors as well as positive affirmations - the desire to avoid wishing cancer on themselves and therefore not entertaining thoughts that they might be at substantial risk 34. Another potential contributor to low perceived susceptibility may be the much lower association between family history of cancer and cancer risk reported by racial/ethnic minority respondents compared to their majority counterparts observed in the current study; this is particularly relevant for breast and colorectal cancer. Previous work examining African Americans' communication about family cancer history has found that even individuals who had experienced cancer in their family reported low communication about this topic 35. This lack of open communication may lead to an underestimated awareness - combined with gaps in knowledge about cancer risk (including cancer genetics) culminating in a misjudgment of one's own cancer risk. However, this identifies another important opportunity for educational intervention and improved health communication.

With respect to cancer screening knowledge, racial/ethnic minority participants were significantly less likely to correctly identify guideline-based ages when colorectal cancer and breast cancer screening should begin, consistent with previous findings 36, 37. Several factors, either individually or in combination, likely contribute to the lower cancer screening knowledge among minorities observed here. Factors identified in previous work include overall lower education attainment as well as missed opportunities for counseling and recommendation by providers 38, 39. One of the most likely contributors to lower screening knowledge is decreased health literacy among minority populations, which is an underlying issue for low uptake of many health-related behaviors. Health literacy interventions, including those specific to colorectal and other cancer screenings, have demonstrated success in improving screening knowledge, attitudes, intention to screen, and screening participation 40, 41. Interestingly, in the present study, despite reporting lower knowledge of screening guidelines, racial/ethnic minority respondents were significantly more likely to report engagement in colorectal cancer screening than majority participants, including receiving an FOBT in the past year as well as ever having a colonoscopy. This may indicate compliance with provider recommendations despite lacking personal knowledge of regular screening guidelines. Although racial/ethnic minority participants demonstrated the only statistically significant lower knowledge of when colorectal cancer and breast cancer screening should begin—compared to Non-Hispanic Whites - it is important to note that the majority of respondents in all four subgroups (racial/ethnic minorities, Non-Hispanic Whites, rural, and urban) were unable to correctly identify the ages at which these screenings are recommended to begin. These knowledge gaps across all study subgroups demonstrate the need for improved and increased education regarding cancer early detection in the overall population throughout the state. Additionally, although most respondents reported receipt of colonoscopy and/or mammogram, the rates of those who received these screening exams within the timeframe of recommended guidelines were under 20% for all four subgroups. The lack of appropriate adherence reported here indicates the need for additional education and initiatives to promote regular screening practices as well as the potential need for interventions addressing barriers to early detection such as limited access and cost.

In generalizing these findings, it is important to acknowledge the limitations of this study for which the primary weakness is the modest response rate. However, the number of respondents and their distribution between subgroups still provided ample power to detect differences of small to medium magnitude. Additionally, the fact that population-based methods were employed adds further strength. Moreover, if respondent bias were indeed an issue, we would likely observe high levels of knowledge and adherence to recommended screening practices, which we did not.

Our findings demonstrate the significant need for early detection cancer education across all subgroups in Alabama, particularly among racial/ethnic minorities. Efforts are needed to improve individuals' understanding of the importance and benefits of cancer screening such as early detection, improved health outcomes, and increased survival. These strategies should also increase perceived susceptibility where relevant and educate regarding heightened risks due to family history of certain cancers while discouraging fatalistic attitudes. Emphasis should be given to clarifying cancer screening recommendations, particularly taking into account health literacy and cultural competency. Finally, future work should consider other barriers to cancer screening that may be specific to rural and/or minority populations such as limited access to care and transportation. Coordination of these strategies has significant potential to improve cancer screening in the overall population and to reduce cancer mortality and disparities.

## Figures and Tables

**Table 1:** Sample characteristics, overall and by minority and rural/urban status

Characteristic*	Overall (n=85/5)	Minority (n=356)	Non-Hispanic White (n=486)	Significance	Rural County (n=671)	Urban County (n=183)	Significance
Age - X (sd)	63.7 (10.15)	63.5 (9.84)	63.9 (10.39	N.S.	63.7 (10.34)	63.9 (9.47)	N.S.
Female Gender, % (n)	57.0% (486)	35.6 (130)	48.1 (233)	<0.001	58.1 (389)	52.7% (96)	N.S.
**Race/Ethnicity, % (n)**							<0.001
non-Hispanic White	57.7% (486)				27.8% (99)	57.6% (102)	
Hispanic White	1.3% (11)				1.1% (4)	0.6% (1)	
African-American	38.0% (320)				68.3% (243)	40.7% (72)	
Mixed Race/Other	3.0% (25)				2.8% (10)	1.1% (2)	
**Rural/Urban Status, % (n)**				N.S.			
Rural	78.5% (671)	37.9%(253)	62.1%(414)				
Urban	21.4% (183)	41.4%(75)	58.6%(106)				
**Marital Status, % (n)**				<0.001			N.S
Married/stable union	54.1% (461)	45.9 (167)	60.4 (293)		54.9% (367)	51.1% (93)	
**Education, % (n)**				<0.001			0.003
<12 years	13.8% (117)	17.0% (55)	12.6% (61)		14.9% (99)	9.8% (18)	
High school (HS) graduate	29.9% (253)	39.0% (126)	24.0% (116)		31.8% (211)	23.0% (42)	
Post HS education/training	56.3% (477)	44.0% (142)	63.4% (306)		53.2% (353)	67.2 (123)	
**Employment, % (n)**				0.020			<0.001
Employed/Full-time student	33.7% (288)	28.5% (104)	37.2% (181)		30.0% (201)	47.0% (86)	
Retired	37.2% (318)	38.3% (140)	36.6% (178)		37.9% (254)	35.0% (64)	
Disabled	20.7% (177)	24.7% (90)	17.7% (86)		22.7% (152)	13.7% (25)	
Unemployed	8.4% (72)	8.5% (31)	8.4% (41)		9.5% (64)	4.3% (8)	
**Annual Household Income, % (n)**				<0.001			<0.001
<$19,999	36.7% (289)	50.5% (168)	26.5% (121)		38.7% (243)	28.2% (46)	
$20,000-$74,999	39.5% (312)	37.5% (125)	41.0% (187)		40.3% (225)	36.8% (60)	
≥ $75,000	23.8% (188)	12.0% (40)	32.5% (148)		20.8% (130)	35.0% (57)	
**Perceived Income Adequacy, %(n)**				N.S.			0.005
Difficulty	14.4% (122)	15.2% (55)	14.0% (67)		15.5% (103)	10.6% (19)	
Getting by	39.2% (331)	41.7% (151)	37.0% (178)		41.0% (273)	32.4% (58)	
Living comfortably	46.4% (392)	43.1% (156)	49.1% (235)		43.5% (289)	57.0% (102)	
Problems w/ Stable Housing, % (n)	1.3% (11)	1.1% (4)	1.4% (7)	N.S.	1.6% (10)	0% (0)	N.S.
**Problems w/ Food Security, % (n)**				<0.001			0.047
Often	4.6% (39)	7.2% (26)	2.7% (13)		4.8% (32)	3.8% (7)	
Sometimes	13.5% (115)	19.7% (71)	8.8% (43)		15.0% (100)	8.2% (15)	
Never	81.9% (695)	73.1% (264)	88.5% (429)		80.2% (534)	88.0% (160)	
No Healthcare Coverage, % (n)	5.9% (50)	6.1% (42)	5.8% (28)	N.S.	5.6% (28)	7.2% (14)	N.S.
**Primary Healthcare Coverage, %(n)**				0.005			N.S.
Medicare	44.1% (347)	45.6% (152)	42.8% (194)		44.7 (278)	41.2% (68)	
Plan through employer/union	36.4% (287)	29.7% (99)	41.1% (186)		350.0 (218	41.8% (69)	
Medicaid	7.0% (55)	9.2% (30)	5.5% (25)		7.6% (47)	4.8% (8)	
Tricare, VA, or military	4.6% (36)	6.1% (20)	3.5% (16)		4.7% (29)	4.2% (7)	
Privately purchased	4.4% (35)	4.2% (14)	4.6% (21)		4.7% (29)	3.6% (6)	
Other	3.6% (28)	5.2% (17)	2.4% (11)		3.4% (21)	4.2% (7)	
No place to go when sick, % (n)	3.4% (29)	3.6% (13)	3.3% (16)	N.S.	3.0% (20)	4.9% (9)	N.S.
**Healthcare Facility Used, % (n)**				<0.001			N.S.
Doctor's clinic	95.3% (812)	93.9% (341)	96.3% (467)		95.7% (639)	94.0% (172)	
Hospital	2.9% (25)	5.0% (18)	1.4% (7)		2.5% (17)	4.4% (8)	
Other	1.8% (15)	1.1% (4)	2.3% (11)		1.8% (12)	1.6% (3)	
Previous Cancer Diagnoses, % (n)	25.0% (213)	19.2% (70)	29.5% (143)	0.001	25.1% (168)	24.7% (45)	N.S.
Access to Internet, % (n)	64.9% (550)	53.9% (194)	73.0% (354)	<0.001	62.2% (414)	74.6% (135)	0.002

* missing data did not differ by minority or urban/rural status, total counts are as follows: age=16; race=6; ethnicity=19; rural/urban status=1 (homeless); education=8; income=66; income adequacy=10; stable housing=3; food security=6; health care coverage=8; source of health care coverage=67; place to go when sick=3; healthcare facility used = 3; previous cancer diagnosis=3; and access to the internet=7.

**Table 2:** Differences in cancer beliefs, knowledge and practices related to cancer screening by minority and rural/urban status

	Minority (n=356)	Non-Hispanic White (n=486)	Significance	Rural County (n=671)	Urban County (n=183)	Significance
**Beliefs related to cancer screening**						
*When I think about cancer, I automatically think of death*			0.015			N.S.
Strongly Disagree	19.3% (69)	12.0% (58)		14.0% (93)	19.4% (35)	
Somewhat Disagree	19.8% (71)	24.4% (118)		22.0% (146)	23.3% (42)	
Somewhat Agree	33.0% (118)	37.3% (180)		35.2% (234)	37.2% (67)	
Strongly Agree	27.9% (100)	26.3% (127)		28.8% (191)	20.0% (36)	
*I'd rather not know about my chance of getting cancer*			<0.001			N.S.
Strongly Disagree	46.6% (165)	34.5% (165)		39.1% (257)	40.4% (72)	
Somewhat Disagree	18.1% (64)	27.0% (129)		23.6% (155)	22.5% (40)	
Somewhat Agree	19.5% (69)	25.1% (120)		22.7% (149)	23.0% (41)	
Strongly Agree	13.6% (48)	15.8% (56)		14.6% (96)	14.0% (25)	
*Compared to others your age, how likely are you to get cancer in your lifetime?*			<0.001			0.016
Much less likely	10.2% (37)	4.6% (22)		5.9% (39)	11.0% (20)	
Less likely	25.0% (91)	14.1% (68)		18.2% (121	20.9% (38)	
About the same	45.1% (164)	52.8% (254)		49.1% (327)	51.1% (93)	
More likely	15.4% (56)	19.3% (93)		19.4% (129)	11.0% (20)	
Much more likely	4.4% (16)	9.1% (44)		7.5% (50)	6.0% (11)	
**Knowledge related to cancer screening**						
A family history of cancer increases a person's chances of getting cancer			<0.001			0.021
Not at All	12.1% (44)	4.1% (20)		7.6% (51)	7.7% (14)	
A little	45.7% (166)	37.8% (182)		38.9% (259)	50.0% (91)	
A lot	42.1% (153)	58.1% (280)		53.5% (356)	42.3% (77)	
Correctly identify the age at which colorectal screening should begin	34.5% (126)	47.9% (233)	<0.001	41.4% (278)	44.3% (81)	N.S.
Correctly identify the age at which mammography screening should begin	36.7% (134)	49.6% (241)	<0.001	44.0% (295)	43.2% (79)	N.S.
Awareness of a test for lung cancer screening?	32.4% (116)	23.6% (113)	0.005	27.2% (180)	27.9% (50)	N.S.
Cancer screening practices*						
Receipt of a fecal occult blood test	40.1% (132)	35.4% (163)	N.S.	37.2% (230)	37.6% (65)	N.S.
If yes, was it in the past year?	17.2% (59)	12.2% (57)	0.046	15.3% (97)	10.8% (19)	N.S.
Receipt of a colonoscopy	82.1% (271)	76.1% (350)	0.041	77.5% (478)	82.8%(144)	N.S.
If yes, was it in the past 10 years?	17.2% (59)	16.7% (78)	N.S.	18.1% (115)	12.5% (22)	N.S.
Receipt of a mammogram?	97.2% (212)	96.6% (229)	N.S.	96.7% (348)	97.9% (92)	N.S.
If yes, was it in the past 2 years?	17.9% (35)	17.1% (35)	N.S.	17.7% (56)	15.5% (13)	N.S.

*Only the cancer screening practices of those eligible for the test are shown.
